# City-wide greenhouse gas emissions of communities nearby the world heritage site of Ayutthaya, Thailand

**DOI:** 10.1038/s41598-022-14036-w

**Published:** 2022-06-13

**Authors:** Paphada Yensukho, Sittisak Sugsaisakon, Suthirat Kittipongvises

**Affiliations:** 1 Inter-Department in Environmental Science Graduate School, Chulalongkorn University, Bangkok, Thailand; 2 Environment Development and Sustainability (EDS) Graduate School, Chulalongkorn University, Bangkok, Thailand; 3 Environmental Research Institute, Chulalongkorn University, Bangkok, Thailand

**Keywords:** Climate sciences, Environmental sciences, Environmental social sciences

## Abstract

Climate change has emerged one of the greatest threats to sustainable development. Cities are a major contributor to high carbon dioxide levels. This research aimed to quantify city-wide GHG emissions and investigate the potential for climate change mitigation in communities near the World Heritage Site (WHS) of Ayutthaya, Thailand via the multi-criteria analytical hierarchy process (AHP). The total city-wide GHG emission of Ayutthaya Municipality in 2018 was 99,137.04 tCO_2_eq (1.93 tCO_2_eq per capita). Energy and waste sectors were the two largest emitters. Pratuchai, the most populated subdistrict and the WHS location, was the largest source of GHGs. However, the cultural heritage site emitted only 0.2% of total GHGs. Based on the IPCC2013 LCA method, residential sector accounted for the largest share (74%), while the WHS contributed only < 1% of total energy-related CO_2_ emissions. If all the Thailand’s Nationally Determined Contribution (NDC) Roadmap are fully implemented in the residential sector, total GHGs would be reduced by 9735.47% tCO_2_eq and 6846.86 tCO_2_eq in 2030. Based on expert interviews, AHP pairwise comparison showed that energy-saving strategies were more preferable than renewable energy technologies. For climate policy initiative, ‘feasibility of implementation’ had the highest AHP weight (0.45) followed by ‘policy feasibility’ (0.39), and ‘environmental performance’ (0.16).

## Introduction

Global urban population has grown rapidly from 751 million in 1950 to 4.2 billion in 2018. As a consequence of rapid population growth, nearly half (54%) of the world’s population live in cities and urban areas. By 2050, this proportion is expected to increase to approximately 68% (from 30% in 1950). Although Asia’s urbanization rate is considerably lower than most regions, it is home to about half (54%) of the global urban population, while Europe and Africa are home to 13% each. Almost 90% of the upward shift in urban population forecasted by 2050 is expected to occur in Asia and Africa regions^1^. Increasing population density in cities brings about risks and challenges to both humans and the environment. In terms of climate change and urbanization, which are inextricable linked^2^, cities have an enormous global carbon footprint. Cities account for over 75% of the global primary energy consumption and emit more than 70% of the total global greenhouse gases (GHGs). From a consumption viewpoint, a recent report by the World Resources Institute^3^ revealed that about 80% of C40 cities contributed the largest share of consumption-based GHG emissions. Over two-thirds of the total emissions, particularly in Europe, North America, and Oceania, are attributable to utilities and housing, transportation, food supply, and government services^3^. Urban metabolism studies^4,5^ focused on flow of energy within a city. For instance, a study of Kennedy et al.^4^ reported that about 4.2–21.5 tons of carbon dioxide were emitted per capita in ten global cities, namely Bangkok, Barcelona, Cape Town, Denver, Geneva, London, Los Angeles, New York City, Prague, and Toronto. The top-ten emitters were all located in surrounding low-density suburbs, and their high GHG emissions were largely attributable to private auto use.

As mentioned above, it is undoubtedly important to pay special attention to the role of cities in minimizing environmental degradation and the negative impacts of increase in number of urban dwellers. Academic research is increasingly focused on city-level CO_2_ emissions due the following reasons: (i) CO_2_ emission databases might reveal various directions towards reducing a city’s emissions, (ii) CO_2_ emissions at the city level help generate improvements in climate policy making, life cycle assessment, and public awareness, (iii) city-level emissions could be more effective than emission intensity where multi-faceted factors are systematically considered^6^. From a policy viewpoint, the concept of low carbon society is probably a major opportunity to achieve climate resilience and promote a more sustainable development. For instance, a study conducted by^7^ addressed the low carbon initiative framework in Thailand as a showcase of Southeast Asian emerging economies because Thailand is considered one of the brightest newly industrialized economies, with vibrant energy- and carbon-emission-intensive growth. Therefore, according to the United Nations Framework Convention on Climate Change (UNFCCC), Thailand’s Intended Nationally Determined Contribution (NDC) aims to reduce GHG emissions by about 20% from the projected business-as-usual (BAU) level by 2030.

In summary, it is clearly apparent that cities must be well-managed, more resilient, and reduce their carbon footprint^2^. Despite its growing importance, however, there are only a few systematic studies in Thailand that focus on estimation of GHG emissions at the city level. City-scale GHG inventory and relevant activity data, particularly in cultural heritage areas, are lacking. Many studies have put more emphasis on emission inventories for specific years at the national scale and sectoral assessment of GHG emissions. Further, most relevant case studies are of provincial capital cities. Therefore, the objectives of this study were to estimate city-scale GHG emission and highlight some mitigation potentials in the WHS of Ayutthaya, Thailand based on a multi-criteria AHP approach. The ultimate expected outcome of this study is to help city policy makers gain a thorough understanding of the important role cities can play in limiting global climate change.

## Methodology

### Case study

**Figure 1:**
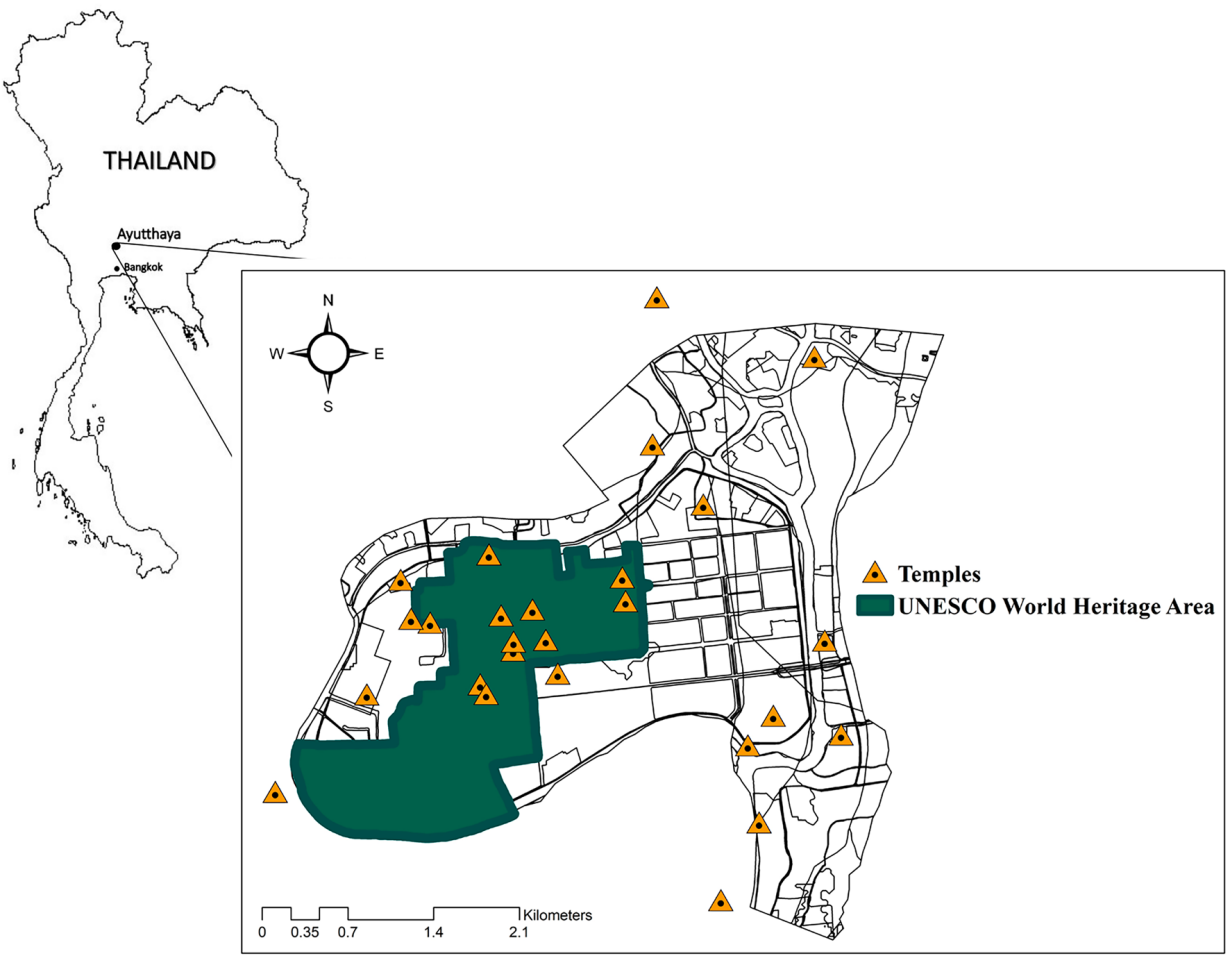
Research case study, Ayutthaya Municipality, Thailand (modified from ^9^).

Based on Thailand’s national economy and the National Economic and Social Development Board report, the five provinces with the highest income (GPP per capita) are Rayong, Chonburi, Bangkok, Phra Nakhorn Si Ayuthaya, and Chachoengsao. This study focuses on the fourth richest city (with a GPP of 14,381.97 USD per capita), Phra Nakhorn Si Ayutthaya, which is an ancient capital in the central region of Thailand. Phra Nakhorn Si Ayutthaya Municipality, which is located approximately 70 km north of Bangkok and has an area of about 14.8 km^2^, was chosen for this research case study. The geomorphological structure of Phra Nakhorn Si Ayutthaya, brought about by the development of towns in the past, has made it into an island surrounded by three rivers—Chao Phraya, Pa sak, and Lop Buri. Demographically, Phra Nakhorn Si Ayutthaya Municipality has a population exceeding 50,000 with approximately 20,220 households (in 2019). It comprises 10 sub-districts: Pratuchai (6344 households), Hua Ro (4312 households), Ho Rattanachai (4185 households), Tha Wasukee (3193 households), Kamung (659 households), Klong Suan Phlu (457 households), Ban Kao (448 households), Huntra (415 households), Klong Sa Bua (116 households), and Khao Rain (91 households). The total area can be divided into the following six land-use types: settlement, governmental area, waterbodies, road/railway, agricultural area, and others. The highest proportion of land (60.5%) is devoted for settlement. As depicted in Fig. 1, by using ArcGIS Pro 2.8.0, approximately 40% (289 ha) of the total island area is protected by the United Nations Educational, Scientific and Cultural Organization (UNESCO) World Heritage Site (WHS), with several cultural heritage aspects including temples, archaeological sites, cultural landscapes, museums, and historical landmarks^8,9^. In 2018, Phra Nakhorn Si Ayutthaya hosted nearly 6.1 million domestic and international travelers.

### Sources and boundaries of city-level GHG emissions

The Global Protocol for Community-Scale Greenhouse Gas Emission Inventories (GPC)^10^ was applied to estimate city-level GHG emissions in this research. Basically, the GPC allows meaningful benchmarking and greater consistency in GHG accounting^11^. By categorizing sources of emissions, the GPC defines three scopes for city inventories. Scope 1 covers GHG emissions from sources within the city boundary, Scope 2 covers GHG emissions attributable to grid-supplied energy, heat, steam and/or cooling within the city boundary, and Scope 3 refers to all other emissions that occur outside the geographical boundary of the city (i.e., out-of-boundary waste and wastewater transportation). The GPC classifies overall GHG emissions into five main sectors: (i) Stationary energy, (ii) Transportation, (iii) Waste, (iv) Industrial Process and Product Use (IPPU), and v) Agriculture, Forestry, and Other Land Use (AFOLU) (Table 1). The geographic boundary of Ayutthaya Municipality served as the boundary for GHG inventory at the city level. All GHG emissions within scopes 1–3 of the GPC were reported (details of scope of GHG emissions are available in supplementary information, Table S1). In terms of source sector, emissions from stationary energy, transportation, AFOLU, and waste in 2018 were reported and expressed as CO_2_ equivalent (CO_2_eq). Meanwhile, emissions from IPPU were excluded as this source does not exist within the case city.

**Table 1:** Sources and scopes of city-level GHG emissions ^10^.

Sectors	Sources and scopes of GHGs emissions		
	Scope 1	Scope 2	Scope 3
**1. Energy sector** (“Energy” section)			
1.1 Residential energy use	Fuel consumption (i.e., Liquefied Petroleum Gas: LPG) (L/month)	Consumption of grid-supplied electricity (kilowatt-hours: kWh)	**–**
1.2 Commercial and institutional buildings energy use	Fuel consumption (i.e., Liquefied Petroleum Gas: LPG) (L/month)	Consumption of grid-supplied electricity (kilowatt-hours: kWh)	**–**
**2. Transport** (“Transport” section)			
On-road transport	Fuel consumption: (i.e., Diesel, Benzene, Gasohol 95, Gasohol 91) (L/month)	**–**	**–**
**3. AFOLU** (“AFOLU” section)	Emissions from rice cultivation	**–**	**–**
**4. Waste** (“Waste” section)	**–**	**–**	Landfill site operation outside the city boundary (Mass of solid waste sent to landfill in inventory year) (tonnes)

### Estimation of GHG emissions

#### Energy

Stationary energy emissions are primarily due to fuel consumption for energy use in residential, commercial, and institutional buildings and facilities (commercial and public buildings such as schools, hospitals, government offices, temples, highway street lighting, and other public facilities) in the city. Specifically, emissions from fuel combustion (Scope 1) and grid-supplied energy consumed (Scope 2) within the city boundary were included. To explore the electricity dataset, real consumption data were obtained from the Provincial Electricity Authority (PEA). A scaled-down method using population indicator^10^ was employed to represent city-wide energy consumption. Equation 1 can be used to estimate CO_2_ emissions from energy use in this sector.

1 $${\text{Energy GHG emissions}} = \sum Energy\,used_{i,j} \times {\text{EF}}_{ij}$$

where energy used is total consumption of electricity (kilowatt-hours; kWh) and each type (*i, j*) of fuel (liter) in the city, and EF is Emissions Factor of each type of energy (*i, j*) (Table S2).

In addition to the application of the GPC, life cycle impact assessment (LCA) was employed to quantify energy-related GHG emissions using a functional unit of ‘1 year of electricity consumption’^12^ in the Ayutthaya Municipality. Using SimaPro 8.3.0, the IPCC 2013 GWP 100a method was followed to evaluate climate change impact as CO_2_ eq.

#### Transport

Ayutthaya Municipality does not have railways, waterways or air transport. Therefore, only ‘on-road’ mode was considered and represented as in-boundary transportation in this research. Following a bottom-up approach, the fuel sold method was employed to estimate GHG emissions from transportation within the city. As shown in Eq. (2), total emissions were quantified via multiplication of quantity of fuel sold by country-specific EFs for each fuel type^13^. Emission factor specific to each type of pollutant was used for estimating transport emission of CH_4_, CO_2_, and N_2_O. Total GHGs was expressed as CO_2_eq.

2 $${\text{GHG emissions}} = \sum {\text{Fuel}} _{j,k } \times {\text{EF}}_{i,j}$$

where Fuel *j,k* = Fuel sales volume or consumption of fuel (*j*) for transport type (*k*); EF*i,j* = Emission factor for compound (*i*) emitted from fuel (*j*).

#### AFOLU

GHG emissions from AFOLU within the city boundary were estimated based on the IPCC Tier 1 method. In terms of in-boundary emissions, only paddy field farming was accounted for in Ayutthaya Municipality. Methane emissions from rice cultivation was quantified using Eqs. 3 and 6, data on annual harvested area, water regimes during cultivation period, crop cultivation period, and soil amendments obtained from the Office of Agricultural Economics (OAE) and Ayutthaya Agricultural Extension Office (DOAE). As noted, GHG emissions from on-road transportation to and from the locations of agriculture and forestry activities were accounted under the transportation sector.

3 $$CH_{4 Rice } = EF_{i } \times t \times A \times 10^{ - 6}$$

where CH_4*Rice*_ is annual methane emissions from rice cultivation (Gg CH_4_/yr), EF_*i*_ is adjusted daily emission factor for a particular harvested area (kg CH_4_/ha/day), A is annual harvested area of rice (ha/yr); *t* is cultivation period of rice (day). In this study, rice cultivation period was 180 days.

4 $$EF_{i} = EF_{c } \times SF_{w} \times SF_{p} \times SF_{0} \times SF_{s,r}$$

where EF_*c*_ is baseline emission factor for continuously flooded fields without organic amendment (1.30 kg CH_4_/ha/day); SF_*w*_ is a scaling factor to account for the differences in water regime during the cultivation period (SF_*w*_ = 0.27 for rain-fed area); SF_*p*_ is a scaling factor to account for the differences in water regime in the pre-season before the cultivation period. SF_*p*_ value of 0.68 was used for non-flooded pre-season of more than 180 days. SF_*o*_ is a scaling factor that should vary for both type and amount of organic amendment applied (Eq. 3). SF_*s,r*_ is a scaling factor for soil type, rice cultivar, and so on. Due to non-availability of soil type and rice cultivar data, SF_s,r_ was not considered in this study.

5 $$SF_{o} = \left( {1 + \sum\nolimits_{i} {ROA_{i} \times CFOA_{i} } } \right)^{0.59}$$

where ROA_*i*_ is the application rate of organic amendment *i*, in dry weight for straw and fresh weight for others (tonne ha^−1^); CFOA_*i*_ is the conversion factor for organic amendment. During the land preparation stage, rice straw was incorporated into soil via ploughing and tilling (> 30 days) before cultivation. CFOA*i* value of 0.29 was chosen in this research.

Total amount of rice straw was estimated from average rice yields and straw to grain ratio (SGR) (Eq. 6), which varied with season, location, and cutting height. An average SGR ratio of 0.75 was chosen.

6 $$Average\,straw\,yield\, \left( \frac{t}{ha} \right) = Average\, production\, yield\, \left( \frac{t}{ha} \right)\times\, SGR\, ratio$$

#### Waste

In this study, CH_4_ emissions associated with waste management activities were defined under Scope 3 because all waste generated in the municipality area were treated at a landfill site outside the city boundary. The IPCC default method was used to estimate methane emissions from solid waste disposal. Specifically, using Eq. (7), methane emissions were estimated based mainly on mass of solid waste sent to landfill annually. All the various types of municipal solid waste collected by municipalities were included in the estimation of degradable organic carbon (DOC) or the organic carbon which is accessible to biochemical decomposition (Eq. 8).

7 $$CH_{4 } Emissions = \left( {MSW_{T} \times MSW_{F} \times MCF \times DOC \times DOC_{F} \times F \times \frac{16}{{12}} - R} \right) \times \left( {1 - OX} \right)$$

where MSW_*T*_ is mass of solid waste sent to landfill in inventory year; MSW_*F*_ is fraction of solid waste disposed to disposal sites; MCF is methane correction factor (fraction); DOC is degradable organic carbon in year of deposition (fraction); DOC_*F*_ is fraction of DOC that is degraded. Tabasaran’s theoretical equation of DOC_*F*_ = 0.014 T + 0.28, where T is temperature is used to determine the value. F is Fraction of methane in landfill gas (IPCC default is 0.5); 16/12 is Stoichiometric ratio between methane and carbon; R is recovered methane; OX is oxidation factor

8 $$DOC = \left( {0.4 \times A} \right) + \left( {0.17 \times B} \right) + \left( {0.15 \times C} \right) + \left( {0.3 \times D} \right)$$

where DOC is degradable organic carbon, A is fraction of municipal solid waste that is paper and textiles, B is fraction of municipal solid waste that is garden waste or other non-food organic putrescibles, C is fraction of municipal solid waste that is food waste, D is fraction of municipal solid waste that is wood or straw.

As previously mentioned, the total emissions of city-wide greenhouse gases of communities nearby the World Heritage Site of Ayutthaya, Thailand were reported in the unit of carbon dioxide-equivalents (CO_2_eq) which is derived by multiplying the total emissions of GHGs by the associated Global Warming Potential (GWP) (i.e., GWP values for 100-year time horizon of IPCC AR4 is 25).

### GHG mitigation options

#### GHG mitigation scenarios

In this research, emissions projections were evaluated under the following two scenarios: (i) business-as-usual (BAU) and (ii) Nationally Determined Contributions- (NDCs-) mitigation plan. Hypothesizing that the current economic trends of the city would continue without technological or structural change, the baseline (BAU) scenario was forecasted from 2018 to 2030. More specifically, mitigation scenarios indicated in Thailand’s NDC were forecasted to the target year (2030) and further compared to the BAU level (2018). Provincial and municipal economic growth rates of 4.13% and 1.12%, respectively, were proposed for forecasting GHG emissions in the case city using Eqs. 9 and 10.

9 $$GPP = \left( {\frac{{GDP_{current\,yr} }}{{GDP_{first\,yr} }}} \right)^{{1/\left( {n - 1} \right)}} - 1$$

where GPP_per capita,n_ = Gross Provincial Product, n = Total number of years

10 $$Economic\,growth\,rate _{municipality} = \frac{{GPP_{per\,capita,\,n} }}{{Population _{municipality,\,n} }}$$

where GPP_*per capita,n*_ = Gross Provincial Product per capita (year n), Population_*n*_ = Total population of municipality (year n).

#### Multi-criteria assessment of climate change mitigation policy

Analytical Hierarchy Process (AHP) was used to prioritize climate change mitigation policy and explore challenges associated with implementing GHG mitigation policy in Ayutthaya Municipality based on multiple criteria analysis. Climate policy analysis employing the AHP involves two steps:

*Step 1*: Selection of climate policy by focusing on mitigation measures indicated in the Thailand’s NDC. Further, the following criteria modified from^14^ were used to assess the preferences for initiating climate policy in a case study: *Environmental performance* (i.e., direct contributions to environmental or climate benefits), *Political feasibility* (i.e., attitude of stakeholders towards cost efficiency and policy possibility/stringency for non-compliance), and *Feasibility of implementation* (i.e., feasibility of climate mitigation measures implementation and financial feasibility).

*Step 2*: Weighting mitigation measure. AHP-pairwise comparison method was employed by interviewing the following experts and decision makers in Ayutthaya Municipality (n = 4): (i) Representative of Ayutthaya Provincial Office for Natural Resources and Environment, Provincial Energy Office, Provincial Electricity Authority, and Ayutthaya Municipality. Theoretically, as presented in Eq. (12), the weighting of each GHG mitigation measure and preference for climate policy implementation (from step 1) was conducted following pairwise comparison matrix with 9-point scale (i.e., less important variables were valued from 1 to 1/9)^15^. All pairwise comparison numerical values were normalized and summed to 1. The consistency ratio (CR) was computed to avoid any incidental judgment in the pairwise comparison matrix (Eqs. 12 and 13). The calculated weighting coefficients are acceptable if the final CR is less than 0.1.

11 $$A = \left[ {aij} \right] = \left\{ {\begin{array}{*{20}c} 1 & {aij \ldots } & {a1n} \\ {1/aij} & 1 & {a2n} \\ {1/a1n} & {1/a2n} & 1 \\ \end{array} } \right\}$$

where *A* = [*a*_*ij*_] is a representation of the expert’s preference for each GHG mitigation measure (*i, j* = 1, 2, …, n)

12 $$CI = \frac{{{\uplambda }\max - { }n}}{n - 1}$$

where λ_max_ is the greatest eigenvalue of the pairwise comparison matrix; *n* is the factor number

13 $$CR = \frac{CI}{{RI}}$$

where CI is the consistency index; RI is the random consistency index (i.e., RI for 9 factors is 1.25).

## Results and discussion

### Total city-wide emissions

**Figure 2:**
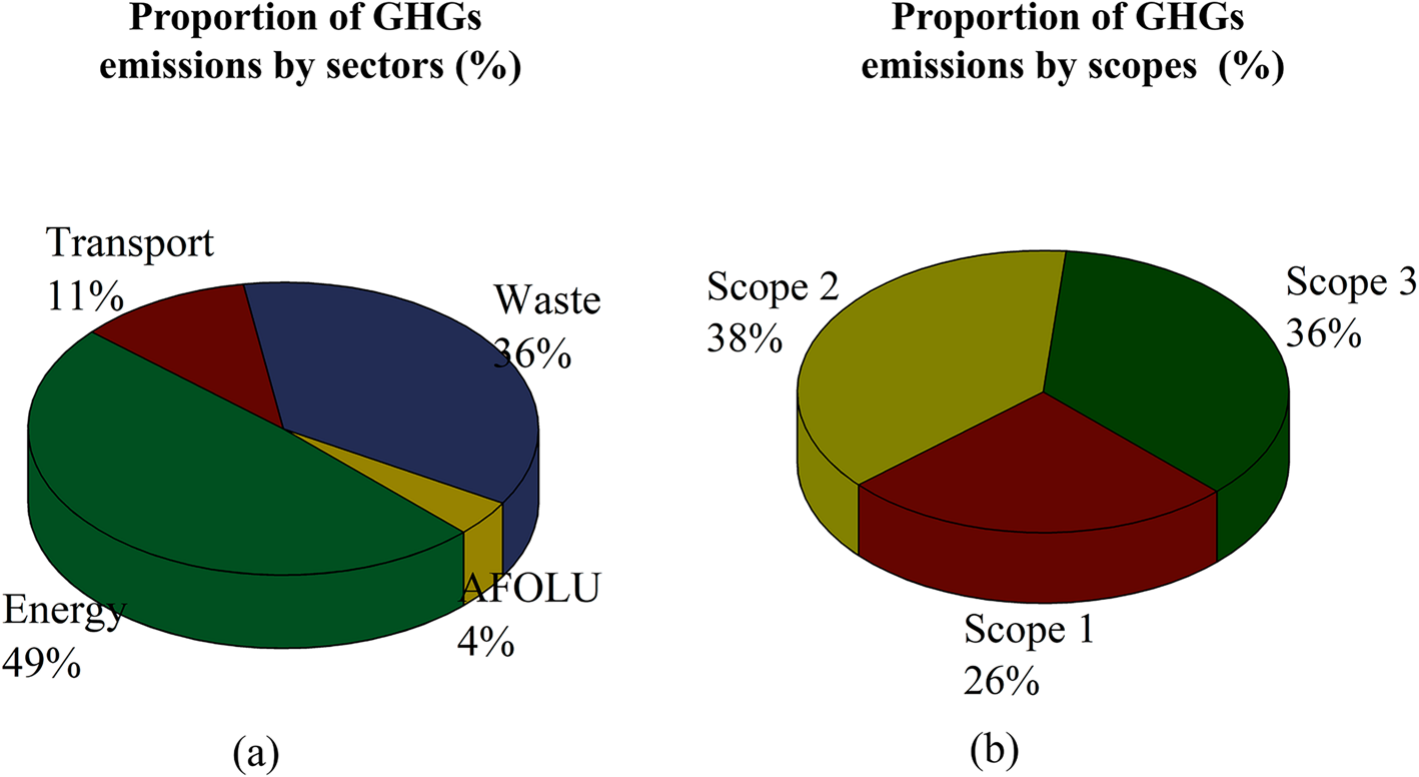
City-wide GHG emissions in Ayutthaya Municipality by (**a**) source of emissions and (**b**) scope of emissions.

In 2018, the total city-wide GHG emissions of Ayutthaya Municipality were approximately 99,137.04 tCO_2_eq. Energy sector was by far the biggest contributor to the total emissions (49%; 48,216.54 tCO_2_eq), followed by the waste sector (36%; 35,659 tCO_2_eq), transportation (11%; 11,191.75 tCO_2_eq), and AFOLU (4%, 4,069.75 tCO_2_eq) (Fig. 2a). Overall, estimated GHG intensity was 1.93 tCO_2_eq per capita. Similarly, a study conducted by the Thailand Greenhouse Gas Management Organization^16^ reported that the annual per capita GHG emissions of some cities (i.e., at the municipal level) in Thailand ranged from 1.28 to 3.74. It should be noted that municipal-level GHG emissions were slightly lower than in denser cities and metro areas. Under such situations, per capita emissions seemed to decline with decline in urban density. For instance, residents in the capital city of Thailand, Bangkok, were responsible for emitting 7.01 tCO_2_eq per capita in 2016^16^. In developed cities like London and New York, per capita emissions were 6.18 (in 2006) and 5.8 (in 2014) tCO_2_eq, respectively^17^. Shan et al.^18^ conducted an inventory of CO_2_ emissions of Chinese cities in 2010 and revealed that Hohhot and Nanping generated the highest (29.67 tCO_2_eq per capita) and lowest CO_2_ emissions per capita (2.38 tCO_2_eq per capita), respectively. On the basis of scope, in this study, indirection emissions from consumption of purchased electricity (Scope 2) in the Ayutthaya Municipality represented the largest source of emissions (38%), followed by Scope 3 and Scope 1 (Fig. 2b).

#### Energy

Energy sector was by far the greatest contributor to the total GHG emissions (49%) in Ayutthaya Municipality. Within this, the residential sector was the primary contributor to GHG emissions (37,672.89 tCO_2_eq; 78%), followed by commercial and governmental organizations (18.49%), and public lighting (i.e., highway street lighting, 3.38%) (Table 2). Among commercial and governmental organizations, hospitals, schools, and hotels located in the municipality released large amounts of GHGs (812.60–4218.84 tCO_2_eq). Compared to other sub-districts, Pratuchai, which is the most populated subdistrict and where the WHS is located, was the largest source of GHG emissions (7797.10 tCO_2_eq). Interestingly, however, the WHS emitted only 206.38 tCO_2_eq (while temples and cultural heritage site emitted 151.63 and 54.74 tCO_2_eq, respectively), accounting for approximately 0.2% of the total city-wide emissions. The results of energy-related GHG emissions obtained using the GPC were similar to those obtained via the LCA approach. Emission results generated using the IPCC2013 GWP 100a method also revealed that the residential sector accounted for a significant share of the total energy-related CO_2_ emissions (74%; 31,866.18 tCO_2_eq). Commercial and governmental organizations emitted almost a quarter (22%; 10,695.76 tCO_2_eq) of this total. Meanwhile, public lighting and the WHS were attributable for only 4% and < 1% of total emissions from energy consumption.

**Table 2:** GHG emissions from the energy sector, by scope and source of emissions.

Sources of GHG emissions	GHG Emissions (tCO_2_eq)				
	Scope 1	Scope 2	Scope 3	Total	%
Residential	10,541.36	27,131.53	**–**	37,672.89	78.13
Commercial and governmental institution	IE	8915.17	**–**	8915.17	18.49
Public illumination	**–**	1628.47	**–**	1628.47	3.38
Total	10,541.36	37,675.18	**–**	48,216.54	100

Relatedly, the WHS was found to account for the lowest GHG emissions using both the GPC and LCA methods. It is possible that most of the ancient buildings at the WHS of Ayutthaya municipality are located outdoors. Many of these historic heritage sites rely mainly on natural light and ventilation for thermal and lighting comfort during the day. In terms of electricity consumption, only lamps are commonly used for lighting, especially at night. However, improving sustainability and energy efficiency in built historic heritage have become high-interest topics among scholars. For instance, European countries with colder climates are most interested in reducing energy consumption in historic buildings^19^. The European Directives showed the potential of the building sector in achieving energy efficiency and reducing carbon emissions. Specifically, study conducted by Serraino and Lucchi^20^ investigated energy retrofit intervention in a castle in Italy and reported that the following criteria must be considered in the implementation of a highly-energy efficient system in historic buildings: achieving building thermal comfort and minimizing CO_2_ emissions and running costs. Some studies have focused on natural lighting design and solar radiation control in heritage buildings^21,22^. A study of Martínez-Molina et al.^19^ also observed that improving indoor climate, energy efficiency, and thermal comfort in historic buildings have become a major issue. Further, Marchi et al.^11^ assessed environmental policies for GHG emission reduction at UNESCO heritage sites of Italy using integrated energy-saving measures and revealed that installation of solar panels on roofs of existing buildings was the most effective environmental policy for decarbonization. GHG emissions equivalent to 17,000 tCO_2_eq per year could be avoided using this measure (57% reduction in GHG emissions in the short term; 10 years).

#### Transportation

In 2018, the volume of GHGs emitted by on-road transport sector of the Ayutthaya Municipality amounted to 11,191.75 tCO_2_eq (11%). The combustion of diesel fuel generated the largest share of emissions (39%) compared to gasohol 91 (37%) and gasohol 95 (24%) for road transport, respectively. It has been suggested that the main factors affecting commuting-related CO_2_ emissions of individuals their and households in local context of Thailand should be more investigated. This finding is in line with Marchi et al.^11^ who reported that diesel is most widely used in the transport sector. By reducing transportation emissions, a study of Li et al.^23^ suggested that many behavioral targeting policies should be implemented in mobility management in cities to reduce private motorized transport that could contribute to a reduction in CO_2_ emissions.

#### AFOLU

Methane emissions from AFOLU within the city boundary were estimated by employing Eqs. 3 and 6. In this research, methane emissions from rice fields were estimated based on emission factor for a particular harvested area of rice, cultivation period of rice (day), and also annual harvested area of rice. In terms of land use, only 2% of the total area of Ayutthaya Municipality (i.e., Khao Rain, Klong Suan Phlu, and Huntra sub-districts) served as agricultural area. All the rice growing areas in the municipality is under rain-fed cultivation. The period of rice cultivation in this study area was about 180 days. The results found that the AFOLU sector of Ayutthaya Municipality emitted approximately 4069.75 tCO_2_eq (accounting for 4% of city-wide emissions). This is within the range of estimated GHG emissions from rice cultivation in other municipalities of Thailand (1.13–6106.75 tCO_2_eq)^16^. In terms of alternatives for mitigating emissions related to rice cultivation water management, Alternate Wetting and Drying (AWD) is a possible option to mitigate methane emission from irrigated rice paddies. A research conducted by Oo et al.^24^ confirmed that yield-scaled GHG emissions from AWD cultivation were significantly lower than from continuous flooding cultivation.

#### Waste

The GPC report^10^ highlighted that city should report GHG emissions from disposal of waste generated within the city boundary, whether treated inside or outside the boundary of city. In this study, the total solid waste sent to landfill in 2018 was 18,130 tonnes, comprising 95.4% of non-food organic waste, 3.3.4% of food waste, and 1.43% of solid waste that is paper and textiles, respectively. The default values of methane correction factor (MCF) of 1 and oxidation factor (OX) of 0.1 for managed landfill were used in estimation of methane emission. In 2018, Ayutthaya Municipality generated 18,130.19 tons of municipal solid waste, all of which was sent to a landfill located outside the city boundary. Within Scope 3 of the GPC, waste sector was the second largest source of GHG emissions, accounting for 35,658.61 tCO_2_eq (36% of the total emission). This result is consistent with other reports in literature; for example, Hoklis and Sharp^25^ reported that in 2009, 338.51 GgCO_2_ eq of methane was emitted from municipal solid waste in Phnom Penh, Cambodia. It was clearly observed in this study that waste disposal activities in tourism cities may result in direct and indirect GHG emissions. Further, previous studies reported that in 2018, tourist areas of Thailand generated approximately 1–2.5 kg of waste per person per day^26,27^. Therefore, behavior of both resident and tourist plays an important role in mitigating climate change, as it determines the solid waste generation rate of the community.

### GHG mitigation scenarios and AHP assessment of climate change mitigation policy

**Figure 3:**
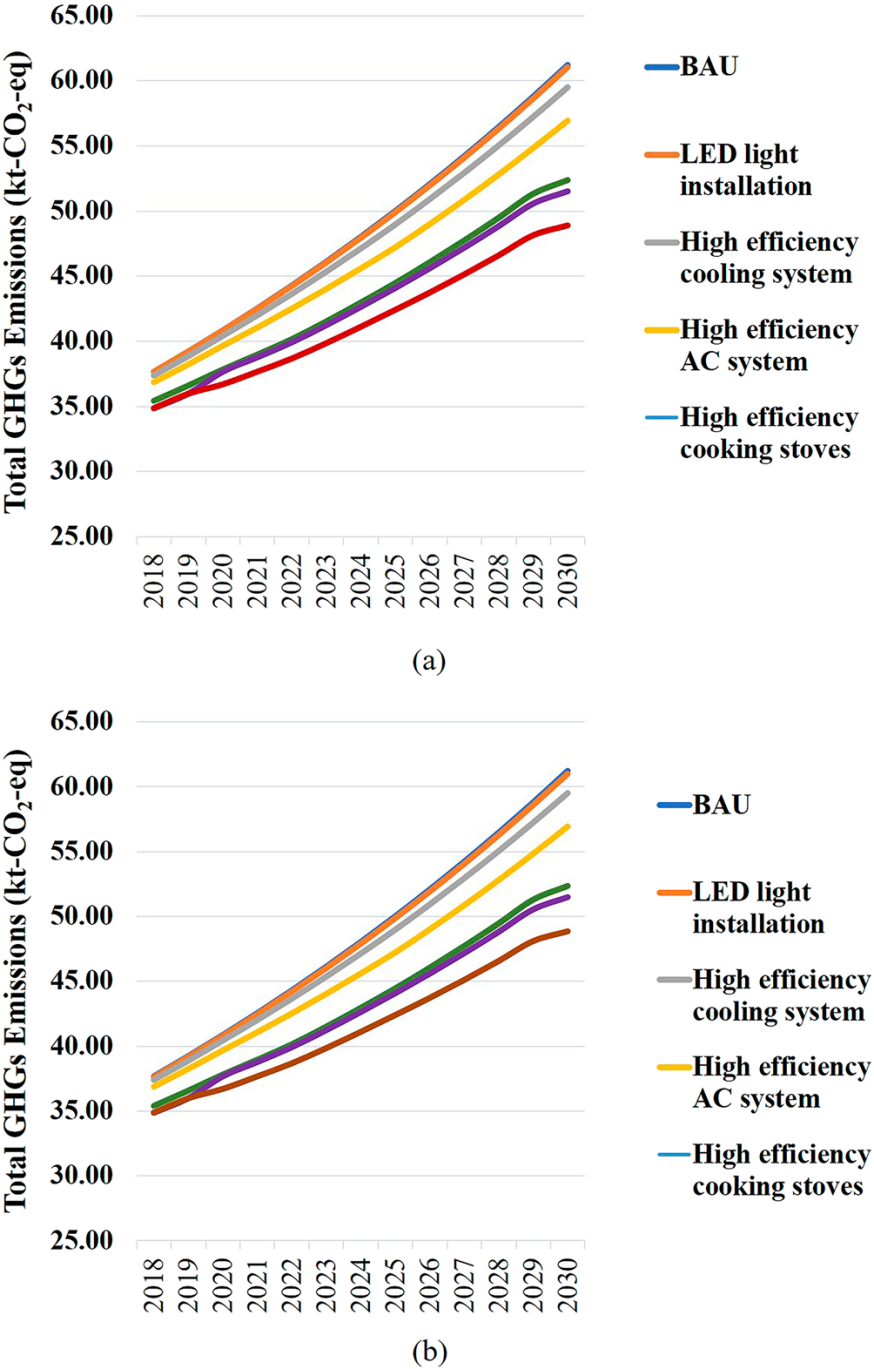
Estimated GHG emissions from the residential sector of Ayutthaya Municipality and mitigation scenarios based on Thailand’s NDC Roadmap assuming (**a**) 4.13% annual provincial economic growth rate and (**b**) 1.12% annual municipality economic growth rate.

Under the BAU scenario, total GHG emissions of Ayutthaya Municipality would increase from 99,137.04 tCO_2_eq in 2018 to 161,123.67 tCO_2_eq and 113,316.63 tCO_2_eq in 2030 in case of forecasted annual economic growth rates of 4.13% and 1.12%, respectively. The residential sector was attributable for the largest proportion of GHG emissions (78%). Under BAU condition, estimated GHG emissions from the residential sector were about 37,672.89 tCO_2_eq. This is projected to increase to 43,056.66 tCO_2_eq and 61,221.79 tCO_2_eq in 2030 at annual economic growth rates of 4.13% and 1.12%, respectively. Potential GHG mitigation options were proposed based primarily on Thailand’s NDC. If all policy interventions as indicated in Thailand’s NDC Roadmap on Climate Change Mitigation (2021–2030) are “fully” implemented in the residential sector, total GHG emissions would be reduced by 9735.47% tCO_2_eq and 6846.86 tCO_2_eq in 2030 assuming 4.13% and 1.12% annual economic growth rates, respectively. These policy interventions include (i) energy-saving measures and strategies and (ii) applying renewable energy in residential buildings. As shown in Fig. 3a,b, the forecast results revealed that applying all energy-saving strategies could provide greater GHG reduction than the implementation of renewable energy policy in the local case study.

According to the judgment on pairwise comparisons of AHP, Tables 3 and 4 revealed that high-efficiency cooling systems should be excluded in the AHP. Installation of LED lighting showed the highest score, followed by improving energy efficiency of air conditioners, and energy efficient appliances and cooking stoves. Establishing renewable energy technologies presented the lowest score in the AHP comparison. In the case of factors that affect climate mitigation policy decisions and implementation, feasibility of implementing mitigation measures (i.e., technical possibilities) presented the highest score (0.45), followed by policy feasibility (0.39) (i.e., policy possibility and cost effectiveness of implementing climate strategies). Surprisingly, environmental performance (i.e., benefits of policy on environment and climate change) showed the lowest score (0.16) in terms of stakeholder’s preference for tackling climate change in the case study. These results are consistent with Heinrich et al.^14^ who revealed that feasibility of implementation was the most important criteria for developing climate change mitigation measures in the power sector. Direct contribution to GHG mitigation as climate benefit was one of the highest AHP criteria, which is inconsistent with this research.

**Table 3:** Overall AHP-weighting of GHG mitigation measures (CR = 0.0542).

GHG mitigation measures	Weight	Ranking
LED lighting	0.395	1
High-efficiency air conditioning system	0.269	2
Energy efficient appliances	0.225	3
High-efficiency cooking stoves	0.048	4
Solar cell power	0.037	5
Biogas energy	0.026	6

**Table 4:** Overall AHP-weighting of the feasibility of climate policy implementation (CR = 0.0904).

Factors		Weight	Ranking
Environmental performance (0.16)	Direct contribution to GHG reduction	0.04	6
	Direct contribution to environmental benefits	0.12	5
Policy feasibility (0.39)	Cost efficiency	0.13	4
	Policy possibility	0.26	2
*Feasibility of implementation* (0.45)	Feasibility of mitigation measure implementation	0.30	1
	Financial feasibility	0.15	3

Overall, as clearly pointed out above, energy consumption in the residential sector was attributable for the largest share of GHG emissions in this research case study. The following recommendations were proposed:

*Aligning city policies for a low-carbon society*: It is strongly suggested that each city in Thailand quantify city-wide CO_2_ emission inventory and their reduction targets. The country’s NDC targets must be linked to city-level policy. Moreover, policy decision makers, local authorities, and all stakeholders should urgently prioritize climate change action policy and mitigation strategies, which had the lowest AHP score, in their local area. Specific detailed information on city-level CO_2_ emissions would be much more meaningful to the decision-making process of local authorities than aggregated information at the national level^28^. However, interestingly, a study conducted by Gouldson et al.^29^ revealed that the absence of multi-level governance arrangements that potentially enable the implementation of low carbon development strategies at the urban level in Asian cities will have global implications for climate change. Moreover, there are still some challenges associated with creating city-level GHG emission inventories such as the use of different methods and approaches and the difficulty of defining city boundary and cross-boundary activities. Further, activities data at the city level are limited and incomparable. It is therefore necessary that data collection for GHG inventory at the city level be as accurate as possible and all uncertainty associated with inventories be avoided.

#### Creating green tourism culture in the WHS

Ayutthaya, a UNESCO WHS, is one of the most famous landmarks and a major tourist attraction of Thailand. The importance of tourism development and GHG emission is now being emphasized by academic scholars. A report by the UNWTO-UNEP-WMP^1^ (2008) revealed that CO_2_ emissions generated by tourism was attributable for approximately 3.9 to 6% of the total global emissions in 2015. Similarly, a report by Katircioglu et al.^30^ found that tourism directly affected both energy consumption and carbon emissions in the long-term economy of Cyprus. In China, research conducted by Meng et al.^31^ assessed carbon emissions of the tourism industry using the Tourism Satellite Account (TSA) and the input–output model and found that the Chinese tourism industry accounted for 2.425–2.489% of the total CO_2_ emissions of all industries in China from 2002 to 2010. Overall, transportation activities accounted for about two-thirds of the total direct emissions, followed by accommodation and food services, and shopping. It should be highlighted that specific information on carbon emissions induced by tourism, especially at the city level in Thailand, are missing. This study strongly suggests that further assessments of carbon emissions from tourism be performed in future research. Further, the impacts of tourism activities and GHG emissions associated with solid waste generation should be urgently explored. Moreover, human and social sustainability of all related environmental and climate change mitigation project should be systematically investigated^32^. 

*Limitations*: It should be noted that there are some limitations associated with this research. For instance, lack of access to data or low reliability of existing data for estimating GHG emissions (i.e., N_2_O inventory) in the AFOLU sector, leading to higher uncertainty associated with these emissions. Specifically, Due to the constraints of this study (i.e., lack of usage statistics for fertilizers in AFOLU sector at the local scale), therefore, only methane emissions were quantified. In the waste sector, lack of updated data on composition of solid waste might have a significant effect on the operational GHG emissions results. Therefore, the critical importance of the life cycle approach and uncertainty/sensitivity analysis should be further considered and accounted in estimating GHG emissions at the city scale.

## Conclusion

Cities are major contributors to the world’s energy-related carbon emissions. However, GHG emission inventories at the city level in Thailand are not well studied. Therefore, the locality of Ayutthaya (a WHS), Ayutthaya Municipality, was selected as a case study. City-wide GHG estimates obtained using the GPC revealed that the most considerable portion of emissions originated from household electricity consumption, especially in Pratuchai, which is the most densely populated subdistrict and the area where the WHS is located. Meanwhile, based on the LCA approach, the WHS contributed only < 1% of the total energy-related CO_2_ emissions. Based on expert interviews and multi-criteria analysis, AHP pairwise comparison revealed that energy-saving strategies was the more preferrable climate mitigation policy compared to renewable energy technologies. Feasibility of implementation and policy feasibility were the most important factors influencing climate mitigation policy in Ayutthaya Municipality. Meanwhile, environmental performance (i.e., climate change co-benefits) was the least preferred factor. Further studies on practical policies for low-carbon development and tourism consumer behavior in the WHS should be urgently investigated.

## Supplementary Information

Supplementary Information.

## Data Availability

The datasets generated and analyzed during the current study are available from the corresponding author on reasonable request.

## References

[1] UNWTO-UNEP-WMO. *Climate Change and Tourism: Responding to Global Challenge*. (The World Tourism Organization and The United Nations Environment Programme: Madrid, Spain, 2008).

[2] Hoornweg D, Sugar L, Gómez CLT (2011). Cities and greenhouse gas emissions: Moving forward. Environ. Urban..

[3] C40 cities. *C40 Cities Climate Leadership Group* https://www.c40.org/ (2021).

[4] Kennedy C, Steinberger J, Gasson B (2009). Greenhouse gas emissions from global cities. Environ. Sci. Technol..

[5] Vande Weghe JR, Kennedy CA (2007). Spatial analysis of residential greenhouse gas emissions in the Toronto Census Metropolitan area. J Ind Ecol..

[6] Tian J, Shan Y, Zheng H (2019). Structural patterns of city-level CO_2_ emissions in Northwest China. J. Clean. Prod..

[7] Criqui, P., Peytral, P. O. & Simon, J. C. Fostering low carbon growth initiatives in Thailand. *Working Papers* (HAL; 2012). https://EconPapers.repec.org/RePEc:hal:wpaper:halshs-00467462.

[8] UNESCO Bangkok Asian and Pacific Regional Bureau for Education. https://bangkok.unesco.org/content/ayutthaya-historical-park (2017).

[9] UNESCO World Heritage Convention. https://whc.unesco.org/en/documents/119855 (2012).

[10] World Resources Institute. *Global Protocol for Community-Scale Greenhouse Gas Emission Inventories: An Accounting and Reporting Standard for Cities* (C40 Cities Climate Leadership Group, & Local Governments for Sustainability (ICLEI), Washington DC, 2014).

[11] Marchi M, Niccolucci V, Pulselli RM (2018). Environmental policies for GHG emissions reduction and energy transition in the medieval historic centre of Siena (Italy): The role of solar energy. J. Clean. Prod..

[12] Stolz P, Frischknecht R, Kessler T, Züger Y (2019). Life cycle assessment of PV-battery systems for a cloakroom and club building in Zurich. Prog. Photovolt..

[13] Thailand Greenhouse Gas Management Organization (TGO) http://thaicarbonlabel.tgo.or.th/download/Emission_Factor_CFO.pdf (2020).

[14] Blechinger PFH, Shah KU (2011). A multi-criteria evaluation of policy instruments for climate change mitigation in the power generation sector of Trinidad and Tobago. Energy Policy.

[15] Saaty T (1980). The Analytic Hierarchy Process.

[16] Thailand Greenhouse Gas Management Organization (TGO). http://www.tgo.or.th/2020/index.php/en/ (2020).

[17] Mayor of London. *Action Today to Protect Tomorrow: The Mayor’s Climate Change Action Plan* (Greater London Authority, 2017).

[18] Shan, Y., Guan, D., Liu, J. & Schroeder, H. CO_2_ Emissions inventory of Chinese cities. Atmos. Chem. Phys. Discuss. 10.5194/acp-2016-176 (2016).

[19] Martínez-Molina A, Tort-Ausina I, Cho S (2016). Energy efficiency and thermal comfort in historic buildings: A review. Renew. Sust. Energ. Rev..

[20] Serraino M, Lucchi E (2017). Energy efficiency, heritage conservation, and landscape integration: The case study of the San Martino Castle in Parella (Turin, Italy). Energy Procedia..

[21] Balocco C, Calzolari R (2008). Natural light design for an ancient building: A case study. J. Cult. Herit..

[22] Cataldo R, De Donno A, De Nunzio G (2005). Integrated methods for analysis of deterioration of cultural heritage: The crypt of cattedrale di Otranto. J. Cult. Herit..

[23] Li P, Zhao P, Brand C (2018). Future energy use and CO_2_ emissions of urban passenger transport in China: A travel behavior and urban form based approach. Appl. Energy..

[24] Oo AZ, Sudo S, Inubushi K, Mano M, Yamamoto A, Ono K, Ravi V (2018). Methane and nitrous oxide emissions from conventional and modified rice cultivation systems in South India. Agric. Ecosyst. Environ..

[25] Hoklis C, Sharp A (2014). Comparison of GHG emission from municipal solid waste management technology in selected cities in Cambodia. Adv. Mat. Res..

[26] Manomaivibool P (2015). Wasteful tourism in developing economy? A present situation and sustainable scenarios. Resour. Conserv. Recycl..

[27] Department of Local Administration of Thailand, Department of Local Administration. http://www.tako.go.th/news/doc_download/a_070818_161026.pdf (2021).

[28] Zheng H, Meng J, Mi Z (2019). Linking city-level input–output table to urban energy footprint: Construction framework and application. J. Ind. Ecol..

[29] Gouldson A, Colenbrander S, Sudmant A (2016). Cities and climate change mitigation: Economic opportunities and governance challenges in Asia. Cities.

[30] Katircioglu ST, Feridun M, Kilinc C (2014). Estimating tourism-induced energy consumption and CO_2_ emissions: The case of Cyprus. Renew. Sustain. Energy Rev..

[31] Meng W, Xu L, Hu B, Zhou J, Wang Z (2016). Quantifying direct and indirect carbon dioxide emissions of the Chinese tourism industry. J. Clean. Prod..

[32] Sugsaisakon S, Kittipongvises S (2021). Citywide energy-related CO2 emissions and sustainability assessment of the development of low-carbon policy in Chiang Mai, Thailand. Sustainability..

