# Supplementary material for: City-wide greenhouse gas emissions of communities nearby the world heritage site of Ayutthaya, Thailand
Source: Sci Rep. 2022 Jun 13;12:9787. doi: 10.1038/s41598-022-14036-w (PMC9192723; doi:10.1038/s41598-022-14036-w)
Supplement: Supplementary file 1 — Supplementary Information. [file 41598_2022_14036_MOESM1_ESM.docx]

1. **Scope of GHG estimation and sources of data collection:**

The Global Protocol for Community-Scale Greenhouse Gas Emission Inventories (GPC) was applied to estimate city-level GHG emissions in this research. Three scopes of GHG emissions were considered as following:

**Table S1** Scope of GHG emissions estimation

| **Scope** | **Related activities/Sources of emissions and data collection** | | |
| --- | --- | --- | --- |
|  | **Scope 1** | **Scope 2** | **Scope 3** |
| 1. **Energy Sector** | | | |
| - 1. Energy use in residential (households) | **Fuel consumption** (i.e., Liquefied Petroleum Gas: LPG)  (L/month)  : data collected from Department of Energy Business, Ministry of Energy and interviewed with local households | **Grid-supplied energy consumed**  (total consumption of electricity (kilowatt-hours; kWh): data collected from Provincial Electricity Authority (PEA) | - |
| - 1. Energy use in Commercial and institutional buildings and facilities (commercial and public buildings such as schools, hospitals, government offices, temples, highway street lighting, and other public facilities) in the city | **Fuel consumption** (i.e. LPG) (L/month)  : data collected from Department of Energy Business, Ministry of Energy and interviewed with local commercial and institutional buildings and facilities | **Grid-supplied energy consumed** (total consumption of electricity (kilowatt-hours; kWh): data collected from PEA | - |
| 1. Transport | | | |
| On-road transport (as Ayutthaya Municipality does not have railways, waterways or air transport) | **Fuel consumption:**  (L/month): Diesel, Benzene, Gasohol 95, Gasohol 91 collected data from petrol station / gas station located in Ayutthaya Municipality. | - | - |
| 1. Waste | - | - | Landfill site operation outside the city boundary (Mass of solid waste sent to landfill in inventory year (tonnes): data collected from Bureau/ Division of Public Health and Environment of Ayutthaya and Ayutthaya Municipality |
| 1. AFOLU | **Emissions from rice cultivation**: Data collected from the Office of Agricultural Economics (OAE) and AyutthayaAgricultural Extension Office (DOAE). | - | - |

1. **Emission factors (EFs) for GHG estimation**

Country-specific EFs for each data activity and source emission were based mainly on Thailand Greenhouse Gas Management Organization (TGO) and some from international organization (i.e. IPCC), as follows:

**Table S2** Emission factors used for GHG estimation

| **Data activity/ source emission** | **EF (kgCO_2_-eq/Unit)** | **Unit** | **Reference sources** |
| --- | --- | --- | --- |
| Electricity | 0.5821 | kWh | Thailand Greenhouse Gas Management Organization (TGO) (2019) |
| Diesel | 2.7446 | L | Thailand Greenhouse Gas Management Organization (TGO) (2019) |
| Gasohol 91 | 2.2376 | L | Thailand Greenhouse Gas Management Organization (TGO) (2019) |
| Gasohol 95 | 2.2376 | L | Thailand Greenhouse Gas Management Organization (TGO) (2019) |
| Liquefied Petroleum Gas | 3.1133 | Kg | Thailand Greenhouse Gas Management Organization (TGO) (2019) |
| EF of continuously ﬂooded ﬁelds without organic amendment | 1.30 | kg CH_4_/ha/day | IPCC (2006) |

1. **GHG Mitigation Scenarios**

Nationally Determined Contributions- (NDCs-) mitigation plan was applied as one of GHG Mitigation Scenarios in this research. As residential sector was the primary contributor to GHG emissions (78%), followed by commercial and governmental organizations (18.49%). According to Office of Natural Resources and Environmental Policy and Planning (ONEP) of Thailand, the following GHG mitigation measures in residential sector indicated by the NDCs were focused (Section 3.2 in manuscript):

Summary information on Thailand’s NDC mitigation measures (Residential sector):

**Energy efficiency improvement**: This measure is divided into the following systems:

- 1. Lighting system
  2. Air conditioning system
  3. Energy-efficient appliances
  4. Other systems (i.e., efficient heater)

**Substitution of renewable energy** (i.e., solar power)
